# Large-Scale Simulation of a Layered Cortical Sheet of Spiking Network Model Using a Tile Partitioning Method

**DOI:** 10.3389/fninf.2019.00071

**Published:** 2019-11-29

**Authors:** Jun Igarashi, Hiroshi Yamaura, Tadashi Yamazaki

**Affiliations:** ^1^Computational Engineering Applications Unit, Head Office for Information Systems and Cybersecurity, RIKEN, Saitama, Japan; ^2^Graduate School of Informatics and Engineering, The University of Electro-Communications, Tokyo, Japan

**Keywords:** large-scale simulation, spiking neural networks, cortex, supercomputer, HPC, parallel computing

## Abstract

One of the grand challenges for computational neuroscience and high-performance computing is computer simulation of a human-scale whole brain model with spiking neurons and synaptic plasticity using supercomputers. To achieve such a simulation, the target network model must be partitioned onto a number of computational nodes, and the sub-network models are executed in parallel while communicating spike information across different nodes. However, it remains unclear how the target network model should be partitioned for efficient computing on next generation of supercomputers. Specifically, reducing the communication of spike information across compute nodes is essential, because of the relatively slower network performance than processor and memory. From the viewpoint of biological features, the cerebral cortex and cerebellum contain 99% of neurons and synapses and form layered sheet structures. Therefore, an efficient method to split the network should exploit the layered sheet structures. In this study, we indicate that a tile partitioning method leads to efficient communication. To demonstrate it, a simulation software called MONET (Millefeuille-like Organization NEural neTwork simulator) that partitions a network model as described above was developed. The MONET simulator was implemented on the Japanese flagship supercomputer K, which is composed of 82,944 computational nodes. We examined a performance of calculation, communication and memory consumption in the tile partitioning method for a cortical model with realistic anatomical and physiological parameters. The result showed that the tile partitioning method drastically reduced communication data amount by replacing network communication with DRAM access and sharing the communication data with neighboring neurons. We confirmed the scalability and efficiency of the tile partitioning method on up to 63,504 compute nodes of the K computer for the cortical model. In the companion paper by Yamaura et al., the performance for a cerebellar model was examined. These results suggest that the tile partitioning method will have advantage for a human-scale whole-brain simulation on exascale computers.

## Introduction

The human brain consists of about ten to the 11th power of neurons and ten to the fifteenth power of synapses (Herculano-Houzel, 2009). In the brain, dozens to hundreds of functional regions exist, and the types of neurons and synapses and the number of connections per neurons differ across regions. To identify these quantities, large-scale measurement techniques have been suggested, including optogenetics (Deisseroth, 2015), connectome analysis (Hunnicutt et al., 2014; Oh et al., 2014; Zingg et al., 2014; Glasser et al., 2016), functional magnetic resonance imaging (Buckner et al., 2011; Yeo et al., 2011), and electroencephalograms (Parvizi and Kastner, 2018; Pesaran et al., 2018). These technical advancements lead to the construction of a realistic human-scale whole brain model using supercomputers. Such large-scale simulations are useful for understanding information processing mechanisms by interactions among brain regions such as cerebral cortical areas (Markram et al., 2015), cerebellum, and basal ganglia, and developmental mechanism of brain disease such as epilepsy, Parkinson’s disease (Moren et al., 2019), Alzheimer disease, and depression, which occur due to interactions among wide areas in the brain. The computational performance of supercomputers has been increased exponentially since their inception and will soon reach 1 exa FLOPS in 2020s, that is, 10th to the 20th power of floating point operations per second. This computational power will allow us to simulate a human-scale brain model with realistic anatomical and physiological parameters.

To date, computer simulations of large-scale brain models using spiking neurons have been widely performed (Izhikevich and Edelman, 2008; Ananthanarayanan et al., 2009; Igarashi et al., 2011; Kozloski and Wagner, 2011; Helias et al., 2012; Yamazaki and Igarashi, 2013; Kunkel et al., 2014; Markram et al., 2015; Jordan et al., 2018; Yamazaki et al., 2019). Dedicated semiconductor chips called neuromorphic chip have been developed for large-scale simulation of spiking neural network (Jin et al., 2008; Schemmel et al., 2010; Furber et al., 2013). In the large-scale simulations, the network communication, the data transfer across computer nodes, has been becoming serious problem with increase in the network size. Modern supercomputers consist of clusters of nodes. For example, the Japanese flagship supercomputer K consists of 82,499 computer nodes connected by a network (Miyazaki et al., 2012). On such a supercomputer, neurons and synapses in a neural network are assigned to multiple computer nodes for parallel processing. When a neuron sends a spike to another neuron, the spike information is transmitted from the source node to the receptor nodes. As the number of spikes exchanged increases, the network communication can become a bottleneck.

In the computer simulation of a large-scale Brunel network model on the K computer, the problem of network communication had occurred at around maximum numbers of compute nodes, and it have been overcome by changing communication function from “MPI_Allgather” to “MPI_AlltoAll” (Helias et al., 2012; Kunkel et al., 2014; Jordan et al., 2018). These studies addressed the computational performance for the worst-case scenario of cortical network which had random connections without spatial organization.

In contrast to the Brunel model with random connections on modern supercomputer, large-scale simulation of spatially organized models on next generation supercomputers leads to two different issues related to network communication.

The first issue is an application of a spatial partitioning method which splits neural networks into sub-networks and assigns them to compute nodes. The cerebral cortex and cerebellum which contain 99% of the neurons and synapses in the brain (Herculano-Houzel, 2009) form layered sheet type structure. The connections tend to be formed in limited spatial extent in the spatial organization. In large-scale simulation of the models with the spatial organization, there is possibility that spatial partitioning method effectively works for reducing the communication data amount.

The second issue is that growth of the network bandwidth is relatively much smaller than those of computational performance and memory bandwidth in next generation supercomputers. For example, the computational performance, memory bandwidth, and network bandwidth per compute node of the next generation of Japanese supercomputer Fugaku released around 2021 is going to be about 20 times, 10 times, and twice greater than those of the K computer released in 2011, respectively (Ajima et al., 2018). The relatively much smaller improved network bandwidth in next generation supercomputers should require more efficient communication than those on predecessors.

To examine the efficiency of the spatial partitioning method for modern and next generation of supercomputers, in this study, we developed simulation software called MONET (Millefeuille-like Organization NEural neTwork simulator) consisting of Python and C programs. The Python program interprets a model description file in JSON format and generates intermediate files which are fed to C program with hybrid parallelization using OpenMP (OpenMP Architecture Review Board, 2008) and MPI (Message Passing Interface Forum, 2009). The Python and C programs run on multicore computers. We implemented our previous cerebral cortical model (Moren et al., 2019) using the MONET simulator. The model is composed of six layers with realistic neuron numbers and ratios, synapse numbers and density, intracellular parameters, and response properties.

We examined effectiveness of the spatial partitioning method for cortical model using the MONET and the K computer. The results showed that the spatial partitioning method benefit from a spatial model of cortex and hardware specification of current and next generation supercomputers. We successfully simulated 6.04 billion neurons and 24.5 trillion synaptic connections corresponding to 1,073 cm^2^ of the layered cortical sheet using 63,504 computer nodes on the K computer. The computational time was about 350 times slower than real time. We also varied the number of computer nodes with a fixed number of neurons and synapses on a single tile and observed a nearly perfect weak-scaling property, suggesting that the parallelization method will enable a human-scale whole cortical simulation on next generation supercomputers within the next 5 years.

This paper is organized as follows. See section “Materials and Methods” described the details of the MONET simulator and the cerebral cortical model. See section “Key Biological Features of the Brain From the Whole Brain Simulation” discusses the key biological features that next generation simulators should include in human-scale whole brain simulation. See section “Results” shows property of tile partitioning method of a model of the cortex and demonstrates the efficiency and scaling properties of the simulator. See section “Discussion” is devoted to general discussion. A companion paper (Yamaura et al., submitted to the same journal) reports the details of the cerebellar model.

## Materials and Methods

### A Three-Dimensional Model of a Layered Cortical Sheet

To evaluate the effectiveness of the parallelization method for spiking neural networks, a three-dimensional model of a layered cortical sheet was developed on the basis of experimental data from the mouse primary motor cortex (M1) and other cortical regions when data from the M1 were lacking.

**FIGURE 1:**
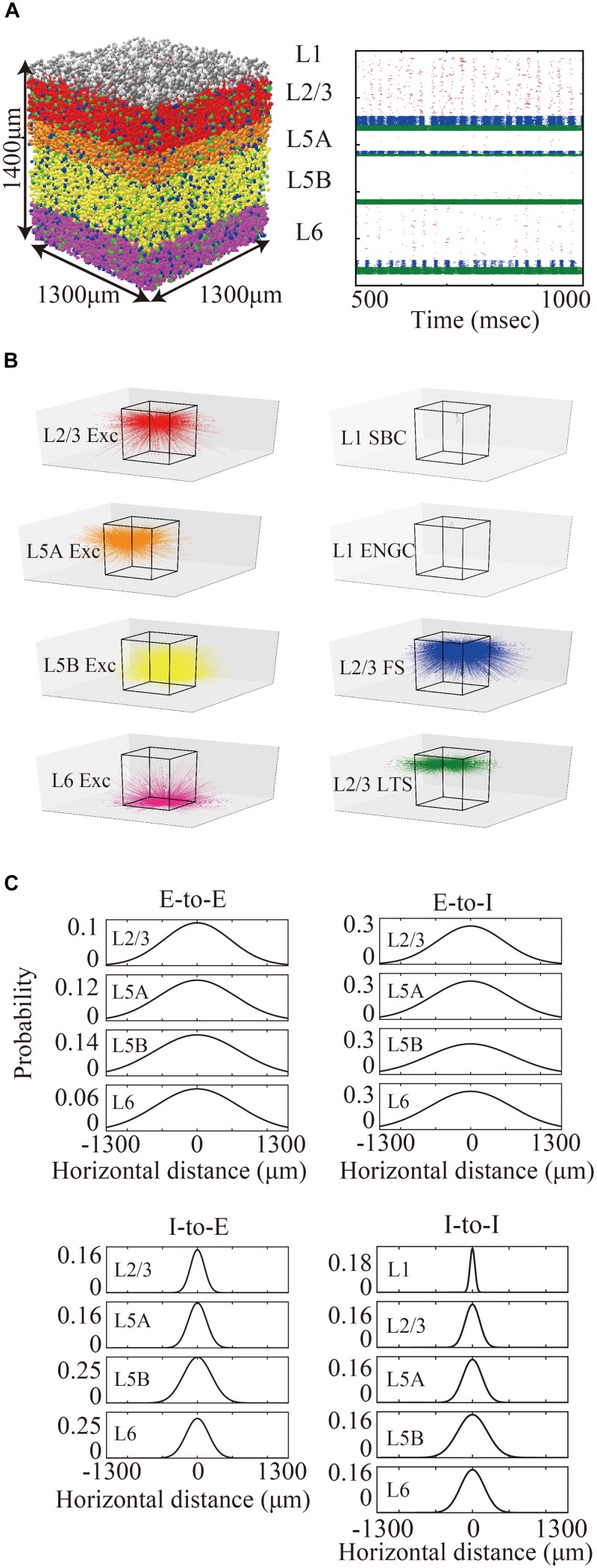
Three-dimensional structure of a layered cortical sheet model. **(A)** 1300 × 1300 × 1400 μm of a layered cortical sheet model. Spheres represent cell positions. Sphere colors: SBC (white), ENGC (gray), L2/3 IT(red), FS (blue), LTS (green), L5A IT (orange), L5B IT (yellow), L6 IT (purple). **(B)** Spike raster plot of the layered cortical sheet. The color code is as in **(A)**. **(C)** In-degree connections of example neurons. The color code is as in **(A)**. The box on the center is 1300 × 1300 × 1400 μm as in **(A)**. **(D)** Connection probability functions of horizontal distance between neurons within layers.

The layered cortical sheet was a cuboid with regular squares on the top and bottom faces (Figure 1A). The direction parallel to the top surface is referred to as horizontal, and the direction perpendicular to the top surfaces is referred to as vertical. The model has six layers consisting of layers 1, 2/3, 5A, 5B, and 6 as classified in M1. The layer thickness and the numbers of neurons in different layers were based on experimental data (Lev and White, 1997; Weiler et al., 2008; Table 1). Fifteen neuron types are included in the model (Table 1).

**TABLE 1:** Neural parameters.

**Layer**	**Neuron type**	**E or I**	***n* Neurons/mm^2^**	**Membrane time constant**	**Ave. connections/neuron**	**Firing rate (Hz)**
1	SBC	I	1259	10	15	0.03
1	ENGC	I	540	10	21	0.02
2/3	IT	E	14659	20	3178	0.06
2/3	PV	I	2290	10	8105	14.7
2/3	SST	I	1374	20	3040	30.7
5A	IT	E	5106	20	3952	0.01
5A	PV	I	774	10	4549	11.5
5A	SST	I	516	20	4215	31.6
5B	IT	E	6072	20	4248	0
5B	PT	E	3036	20	4673	0
5B	PV	I	1822	10	6440	0.05
5B	SST	I	1215	20	5283	58.58
6	IT	E	14102	20	2279	0.01
6	PV	I	1763	10	9908	1.7
6	SST	I	1763	20	5586	16.6

Layer 1 includes only two inhibitory neuron types: single bouquet cells (SBC) and elongated neurogliaform cells (ENGC, Jiang et al., 2013). Layers 2/3, 5A, and 6 have one excitatory neuron type, intratelencephlic neurons (IT), and two inhibitory neuron types, parvalbumin-expressing (PV), and somatostatin-expressing (SST) interneurons (Tremblay et al., 2016). Layer 5B has two excitatory neuron types, IT and pyramidal-tract (PT) neurons (Shepherd, 2013), and two inhibitory neuron types, PV and SST.

The ratio of the numbers of excitatory neurons to inhibitory neurons was set as 4:1 in all layers except for layer 1 Neurons are placed on spatial positions generated by pseudo-random numbers within the space of the layer to which the neuron belongs.

All neuron types are implemented using a leaky integrate-and-fire model. A neuron *i* evolves according to

$$\tau_{m}\,\frac{d\,u_{i}}{d\,t}=-u_{i}+u_{r\,e\,s\,t}+R_{m}\,\left(I_{s\,y\,n,i}\,\left(t\right)+I_{e\,x\,t,i}\right),$$

$$\text{if}\,u\,\left(t\right)=\theta \Rightarrow u\to u_{\text{r}},$$

where *u*_*i*_ is a membrane potential, *u*_*rest*_ is a resting potential, *t*_*m*_ is a membrane time constant, *R*_*m*_ is a membrane resistance, *I_*syn*_,_*i*_* is a synaptic current, *I*_*ext*_,*_*i*_* is an external current, *t* is time, θis a spike threshold, and *u*_*r*_ is a reset value of membrane potential. The synaptic current is described as

$$I_{s\,y\,n,i}\,\left(t\right)=\sum_{j}\sum_{t_{j}^{f}}W_{i\,j}\,g_{s\,y\,n}\,\left(t-t_{j}^{f}\right)\,\left(u_{i}-E_{s\,y\,n,i,j}\right),$$

where *W_*i*__*j*_* is a connection weight from neuron *j* to *i*, *g*_*syn*_ is a time-dependent function of synaptic conductance, *t_*j*_^*f*^* is a spike time from neuron *j*, and *E_*syn*_,_*i,j*_* is reversal potential of synaptic ion channel.

The membrane time constants are 20 ms, except for SBCs, ENGCs, and PVs, which have a time constant of 10 ms. All neurons receive constant bias currents, which follow a normal distribution, to generate spontaneous firing at a low rate, as seen in the cortex in a resting state. The normal distributions have a mean of 10 and standard deviation of 5 for neurons in L1 and excitatory cells and a mean of 18 and standard deviation of 5 for inhibitory neurons in L2/3–6. The average firing rate of total neurons is around 4 Hz, as seen in resting state, with a low rate and irregular firing (Figure 1B).

#### Overview of Experimental Data Used for Modeling

Each combination of presynaptic and postsynaptic neuron types has its own connection parameters. Two types of experiments, laser-scanning photo-stimulation (LSPS) and patch-clamp recording experiments, were used to provide information for establishing appropriate connection settings.

In the following sections, we describe the details of connection settings using the information from the LSPS and patch-clamp recording experiments.

#### Pre-processing of Information About LSPS

The LSPS responses of connections from excitatory to excitatory (E-to-E) cells were reported by Weiler et al. (2008), connections from inhibitory to excitatory (I-to-E) cells by Kätzel et al. (2011), and connections from excitatory to inhibitory (E-to-I) cells by Apicella et al. (2012). These responses are the sums of synaptic inputs generated by a presynaptic neural population stimulated by laser spots, in which the signal values reflect the number of connections, synapse conductance, and density of the stimulated presynaptic neurons. To remove the contribution of the neural densities, the LSPS responses were divided by the neural densities of the presynaptic neurons (Lev and White, 1997). Matrices of processed LSPS response values were used for setting the relative magnitude of connection probabilities of the connections.

#### Details of LSPS Experimental Data

Reports about E-to-E connections and I-to-E connections provided data pertaining to the spatial extent of connections within the layer where the presynaptic neurons are located, and matrices of LSPS responses for all combinations of presynaptic and postsynaptic layers (Weiler et al., 2008; Kätzel et al., 2011). The spatial extent and relative connection probability of E-to-E and I-to-E connections were set in the setting parameters using the above-mentioned information.

There is no comprehensive information available about the E-to-I and I-to-I connections for all layers. Assuming that the axons of presynaptic neurons innervate postsynaptic excitatory and inhibitory cells in a similar spatial range, the spatial extents of the E-to-E and I-to-E connections were used for setting the parameters of E-to-I and I-to-I connections, respectively, except for the E-to-I connections of layers 5A and 5B for which information is available.

PVs and SSTs have different parameter settings for E-to-I connections. Given that the PVs tend to receive excitatory connections as do neighboring excitatory cells (Xu and Callaway, 2009; Apicella et al., 2012; Avermann et al., 2012; Xue et al., 2014), information about E-to-E connections can be applied to the E-to-I connections of PVs in layers 2/3 and 6.

Information about E-to-I connections of PVs within layer 2/3 is set using data from the connections of SSTs within layer 2/3, considering the similarities found in patch-clamp recording experiments (Avermann et al., 2012; Pala and Petersen, 2015). The PVs and SSTs in layers 5A and 5B have individual parameter values for the E-to-I connections based on data from LSPS experiments (Apicella et al., 2012).

In the case of the I-to-I connections, only specific pairs of neuron types, such as PVs to PVs and SSTs to PVs (Pfeffer et al., 2013), have connections. The information about I-to-E connections was applied to specific I-to-I connections.

The SBCs and ENGCs in layer 1 were connected according to the spatial information and morphological features reported by Jiang et al. (2013) and Lee et al. (2015).

#### Setting of Connection Probability Using Gaussian Functions

Connection probabilities were set using two-dimensional Gaussian functions for distance between neurons, where the distance is horizontal distance. During the pre-processing of information from LSPS experiments, information about the variances of the Gaussian functions were derived by Gaussian fitting of extracted data from previous reports. The spatial extent of *trans-*laminar connections were set to be the same as those of the intra-laminar connections, except for the E-to-I connections of PVs and SSTs in layers 5A and 5B, for which specific information about *trans-*laminar connections are available (Apicella et al., 2012). The cutoff distance of the Gaussian function was 1300 μm from the center of the peak that corresponds to two standard deviations of the widest Gaussian function of all connections.

Data from LSPS and patch-clamp recording experiments were used for setting the center of the peak of the Gaussian functions. Data from LSPS experiments were used for establishing the relative levels of the centers of the peaks of different connection types. Data from patch-clamp recording experiments provided the ranges of the absolute levels.

The maximum values of the centers of the peaks of the Gaussian functions for E-to-E, E-to-I, I-to-E, and I-to-I connections were set to 0.12, 0.25, 0.25, and 0.25, respectively, based on data from patch-clamp recording experiments, which provide information from neighboring neuron pairs in the somatosensory cortex (Lefort et al., 2009; Avermann et al., 2012; Pala and Petersen, 2015) and the visual cortex (Pfeffer et al., 2013). According to these reports, the maximum probabilities of E-to-I, I-to-E, and I-to-I connections (55∼60%) are more than twice as high as those of E-to-E connections (∼25%). Figure 1D shows the connection probability functions of E-to-I, I-to-E, and I-to-I intra-laminar connections.

The connection weights of E-to-E connections follow a log-normal distribution (Song et al., 2005; Lefort et al., 2009). The parameter of the exponent of the distribution is −0.72. E-to-I, I-to-E, and I-to-I had constant values. The connection weights were 0.42 for connections between excitatory neurons and PV, 0.19 for excitatory to SST, 2.00 for PV or SST to excitatory neurons, 1.12 for PV to PV, 0.74 for PV to SST, 1.00 for ENGC to other neurons, and 0.80 for SBC to inhibitory neurons. The spatial distribution of connection weights was globally homogeneous. Time changes in synaptic conductance were described using an alpha function. The time constants of excitatory and inhibitory synaptic conductance were 2 ms. The reversal potential of excitatory and inhibitory connections were 0 and -70 mv, respectively.

The total number of cells and connections per 1 mm^2^ cortical sheet were 56,291 and 212 million, respectively. Figure 1C shows spatial extent of in-degree connections of example cells. The average numbers of connections per cell were 3781. For more information, see Table 1.

### MONET: In-House Spiking Neural Network Simulator for Tile Partitioning

To test the tile partitioning method for realistic spiking neural network models on parallel computers, we developed an in-house neural network simulator, called MONET (Millefeille-like Organization NEural neTwork simulator). The binary executable files of the MONET are available at https://github.com/junigarashi/MONET.

The MONET simulator partitions the layered sheet types of spiking neural networks into regular square tiles, distributes the tiles to compute nodes, and performs parallel computing with asynchronous point-to-point communication using the MPI library for the communication of spike information. MONET runs on a distributed computer system consisting of compute nodes with multicore CPUs. The following sections describe internal processing in MONET with respect to the construction of networks, execution of the simulation, and the connection data structure.

#### Construction of Neurons and Connections in Neural Network

Initially, MONET creates a data structure of neurons and sets parameters for each compute node according to information in intermediate files (see section “Usage Procedure of MONET”). Each compute node sets the positions of the neurons in its assigned tile and shares those positions through MPI communication only with neighboring compute nodes that have candidate postsynaptic neurons. Each compute node makes connections based on the distances between neurons and the connection-setting parameters, including information about the connection probability, connection weight, and signal transmission delay. Information about connections is stored at each compute node with a tile containing postsynaptic neurons.

#### Parallel Computing and Communication in Simulation

Millefeuille-like Organization NEural neTwork simulator carries out parallel computing of simulation of spiking neural networks using a tile partitioning method and asynchronous point-to-point communication.

Spike information is communicated among compute nodes at an interval of a half of the minimum transmission delay. The way of the communication in MONET is an modified version of those used in the simulators NEST and NEURON (Carnevale and Hines, 2006; Gewaltig and Diesmann, 2007). The original communication method transfers spike information using synchronous collective communication at an interval of minimum signal transmission delay in the network and can completely keep right communication of spike information among compute nodes in time and reduce communication frequency.

The communication at an interval of a half of the minimum transmission delay in MONET make possible to use asynchronous communication, which allows concurrent execution of the communication of spike information and the numerical calculation of state variables about neurons and synapses.

**FIGURE 2:**
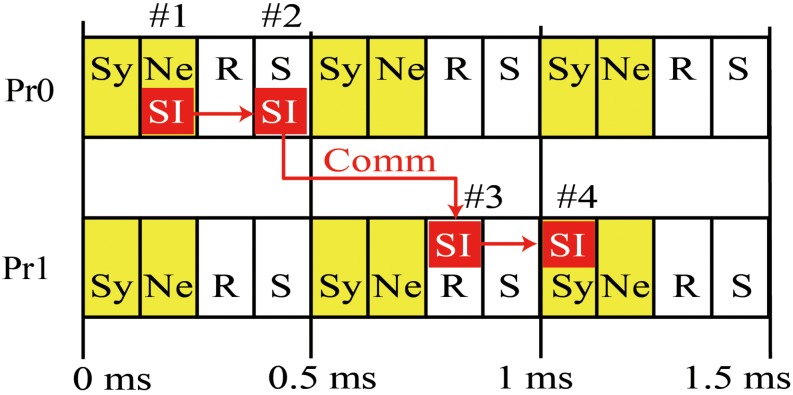
Concurrent execution of communication and calculation. Diagram shows order of calculation and communication of two MPI processes. (#1) MPI Process 0 (Pr0) stores spike information at calculation of state variables of neurons for 0–0.5 ms. (#2) MPI Pr0 sends it to Process 1 (Pr1). (#3) Pr 1 receives it at the step of 0.5 ms and uses it in calculation of state variables of synapse at the step of 1–1.5 ms (#4). Therefore, the calculation of synaptic conductance and neurons in 0.5–1 ms overlaps with communication of spike time information (SI) between S by Pr0 during 0–0.5 ms and R by Pr-1 over 0.5–1 ms. Yellow boxes denote numerical calculations. Sy and Ne in the boxes represent the calculations of synapse and neuron, respectively. White boxes denote communication. R and S represent receive and send, respectively. Small red boxes represent SI originating in Pr0. The 0.5 ms interval of calculation and communication is a half of the minimum signal transmission delay.

Figure 2 is a diagram of the order of calculation and communication in a case where there are two MPI processes. Each process first calculates the state variables of the synapses and neurons and stores the resulting spike information. The process then performs asynchronous communications related to the reception of spike information in the previous interval and the sending of spike information in the current interval. The sending of spike information overlaps with numerical calculation in the next interval. The spike information in the current interval generates synaptic conductance after the next interval when half of the minimum signal transmission delay is used as an interval. Therefore, in the example case with 1 ms of signal transmission delay in Figure 2, the spike information generated in 0–0.5 ms reaches the MPI process with postsynaptic neurons within 0.5–1 ms, and calculation of the synapse conductance uses the spike information in 1–1.5 ms.

#### Connection Data Structure

In realistic modeling of the cortex and cerebellum using single-compartment leaky integrate-and-fire model, connections account for most of the calculation, memory access, and memory consumption in a simulation.

**FIGURE 3:**
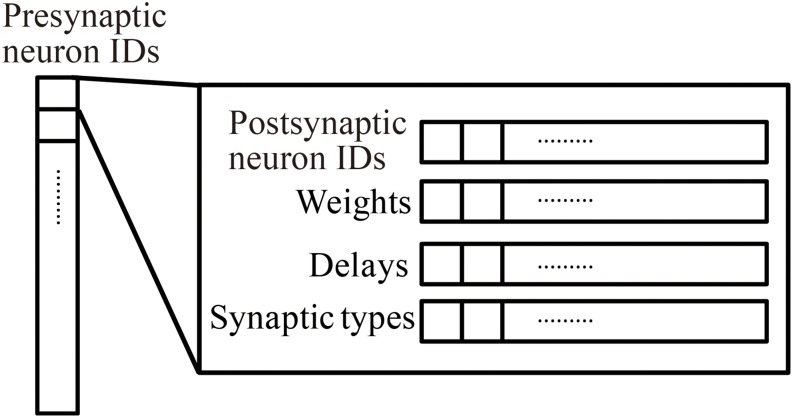
Hierarchical connection data structure. Vertical array in the left-hand side represents an array of presynaptic neuron IDs. The individual element is a structure containing four arrays of postsynaptic neuron information, postsynaptic neuron IDs, weights, delays, and synapse types.

Millefeuille-like Organization NEural neTwork simulator adopts a hierarchical data structure of connections (Figure 3). The indices of the array at the top level represent presynaptic neuron IDs. The structures at the second level contain information about postsynaptic neurons as six arrays with a number of elements corresponding to the number of postsynaptic neurons. The data contents and data sizes of the elements of the six arrays in the structure are postsynaptic neuron IDs (four bytes), weights (four bytes), signal transmission delays (two bytes), synaptic types (two bytes), synaptic plasticity types which is booked for future development (one byte), and a local identifier of the synapse (one byte). The total size of the information per connection is 14 bytes.

When calculating synaptic conductance, the presynaptic neuron IDs initially point to one structure containing information about postsynaptic neurons and connections. A loop through the arrays in the structure performs the calculation of the synaptic conductance of postsynaptic neurons in sequence.

#### Numerical Calculation

A forward Euler method with calculation step of 0.1 ms was used for all numerical calculations pertaining to neurons. The matrix exponential method (Rotter and Diesmann, 1999) was used for the calculation of the time evolution of synapse conductances. Xorshift was used (Marsaglia, 2015) as algorithm for pseudo-random number generation.

### Usage Procedure of MONET

**FIGURE 4:**
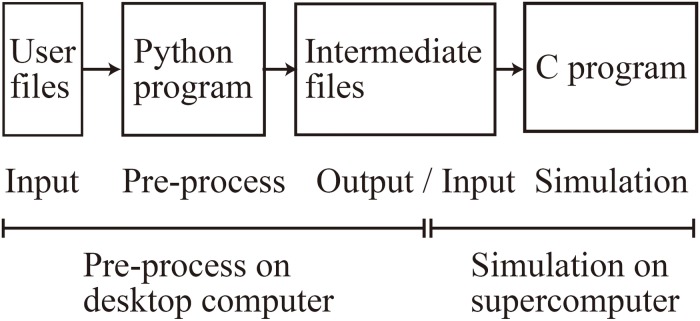
Overview of the MONET simulator. The procedures consist of two phases: pre-processing on a desktop computer and simulation on a supercomputer.

When using MONET, users take three steps: (1) prepare user-defined files in JSON format, (2) make intermediate files fed to compute nodes using a MONET Python program, and (3) execute the simulation using MONET C program on computers with the intermediate files (Figure 4). The following sections explain how users set up spiking neural network models and their settings for parallel computing using MONET.

#### User-Defined Files in JSON Format

Users define two JSON files: “system.json” describing the parameters used in parallelization and the computational environment and “region_X.json” describing the settings of a neural network (Figure 4). In the two JSON files, users describe the setting information using a hierarchical data structure.

**FIGURE 5:**
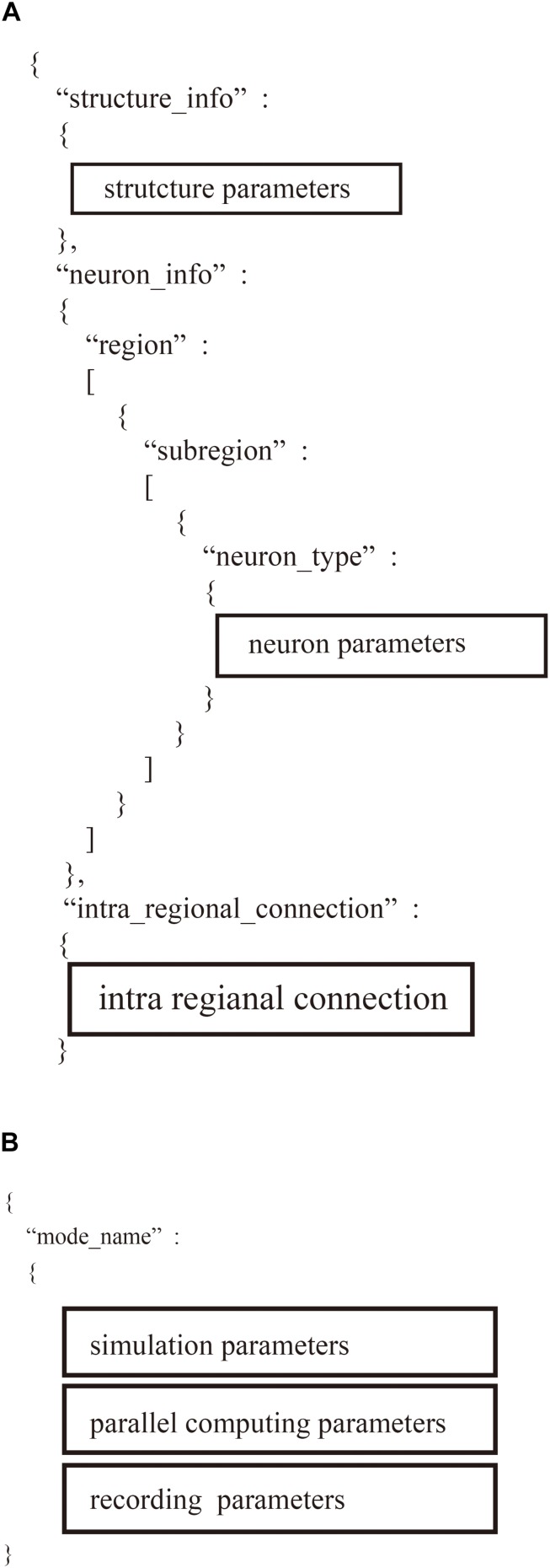
Hierarchical data structure in two user-defined files. **(A)** Region_X.json describes information of neural network models including parameters of structure of the model, property of neurons, and connections. **(B)** “system.json” contains information of settings of simulation, parallel computing, and recordings.

The “system.json” file describes the settings of the simulation: biological time, calculation step size, the number of MPI processes and OpenMP threads, the seed for the pseudo-random numbers, amount of memory per compute node, and recording of neural activity (Figure 5A).

The “region_X.json” file has three major sections (Figure 5B): (1) structure of the neural network, (2) parameters of the model neurons, and (3) settings of the intra-regional connections.

#### MONET Python Program and Intermediate Files

At the second step, users generate intermediate files that are fed to compute nodes by executing a MONET Python program. The program reads two user-defined JSON files and determines which tiles will communicate, based on their positions and spatial extent of communication. The program creates intermediate files for the number of compute nodes. Each intermediate file provides the parameters for simulation settings and neural network of one tile for a compute node.

#### Execution of Simulation by the MONET C Program With Intermediate Files

**FIGURE 6:**
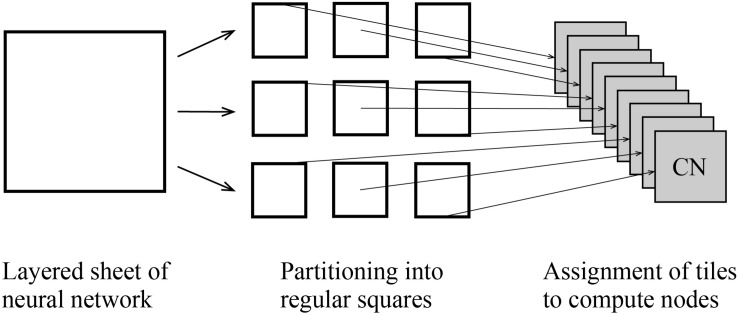
Tile partitioning of the layered sheet model and assignment of the tiles to compute nodes. The layered sheet **(left)** is partitioned into regular square tiles **(middle)** and assigned to compute nodes **(right)**.

At the third step, users execute a MONET C program to simulate spiking neural networks on computers with intermediate files fed to compute nodes. MONET performs simulation by hybrid parallelization using MPI and OpenMP. One MPI process runs on one CPU and one OpenMP thread runs on one CPU core. One tile is assigned to one MPI process (Figure 6). After simulation, the MONET output result files consisting of neural activity, positions of neurons, and elapsed times of calculation.

### Computational Environment

#### Software Environment

The C program in MONET was developed with MPI library functions and OpenMP directives and compiled using Fujitus C/C++ compiler (fccpx) version 1.2.0. The Python program in MONET was developed using Python version 2.7.12. The MONET Python and C program runs in a Linux environment.

#### Hardware Environment

The MONET C program runs on the K computer at the RIKEN Center for Computational Science in Kobe, Japan (Miyazaki et al., 2012). The K computer is a Japanese national supercomputer which was developed with about 72 million dollar. There are opportunities for domestic and foreign researchers to use the K computer if their application is accepted. The K computer has 82,944 compute nodes, each of which has an 8-core SPARC VIII fx processor operating at 2 GHz and 16 GB of DRAM with 64 GB/s of memory bandwidth. The total theoretical computational performance is 10.62 PFLOPS, and the total amount of memory is 1.26 PB. The compute nodes are connected by a six-dimensional mesh/tours network called Tofu interconnect. Each compute node has 10 links in the Tofu interconnect. The theoretical bandwidth of one link is 5 GB/s.

The MONET Python program runs on a workstation, a Dell precision T7810 which has two eight-core CPUs running at 2.1 GHz (Intel Xeon E5 2620) and 128GB DRAM.

## Key Biological Features of the Brain From the Whole Brain Simulation

To achieve efficient calculation using high-performance computing, it is necessary to identify the main part of the code in terms of amount of computation and the bottlenecks in the calculation. Parallelization and acceleration should be performed so that computational loads are equally distributed to the compute nodes of a parallel computer, and efficient communication is performed among compute nodes. Here, we outline three major anatomical and physiological features of the brain from the viewpoint of the parallel computing of whole brain models and consider how to parallelize the model.

### Major Aspect of the Brain Consuming Computational Resources

The first key feature is about the population of neurons in the brain. The numbers of neurons and synapses determines the total amount of calculation and memory consumption in large-scale spiking neural networks (Izhikevich et al., 2004; Izhikevich and Edelman, 2008; Ananthanarayanan et al., 2009; Helias et al., 2012; Kunkel et al., 2014; Jordan et al., 2018; Yamazaki et al., 2019). In the mammalian brain, the cortex and cerebellum contain most of the neurons in the brain: 19% in the cortex and 80% in the cerebellum (Herculano-Houzel, 2009). The cortex and cerebellum are also estimated to include most of the synapses in the brain, because they account for most of the brain volume: 82% in the cortex and 10% in the cerebellum (Herculano-Houzel, 2009). Therefore, the cortex and cerebellum require most of the computation in spiking neural networks of whole brain models. Although the other remaining parts of the brain are undoubtedly crucial in information processing, irrespective of the numbers of neurons and volume, we focus on the simulation of the cortex and cerebellum to appreciate the efficiency of parallel computing in the current study.

### Layered Sheet Type Circuitry and Its Partitioning

The second key feature is the circuit structure of the major brain regions in the cortex and cerebellum. The cortex and cerebellum have similar circuit structures, layered two-dimensional sheets, like a Millefeuille (Eccles et al., 1967; Standring, 2016), although they are folded in the brain in a complicated manner. The numbers of layers, neuron types, and neural densities are similar over the entire cortical sheet (Standring, 2016) and cerebellar sheets (Eccles et al., 1967).

In parallel computation of a simulation of the layered sheet types of neural network models, one of the ways to parallelize the model is by spatially partitioning the sheets into tiles. This is called the tile partitioning method and is equivalent to cutting Millefeuille into equal-sized pieces. The tile partitioning of the neural sheets can place a similar amount of neurons and synapses per tile if the quantities are homogeneous over the entire sheet. The equal assignment of neurons and synapses leads to load balancing in parallel computing.

The other form of parallelization is a round-robin partitioning, in which neural elements are evenly assigned to compute nodes in a round-robin manner without maintaining spatial structure. In principle, round-robin partitioning is better than spatial partitioning for load balancing, due to its fine-grained partition. However, the tile partitioning method can also produce load balancing at a similar level to that of the round-robin method if the neural sheets are sufficiently homogeneous.

### Local-Dense and Remote-Sparse Connections and Parallelization

The third key feature is connection patterns, which reflect the communication performance of spike information among the compute nodes. In rodent brains, neurons tend to connect densely with neighboring neurons that are located within around 1 mm in the same brain regions (Weiler et al., 2008; Xu and Callaway, 2009; Fino and Yuste, 2011; Hooks et al., 2011; Kätzel et al., 2011; Packer and Yuste, 2011; Apicella et al., 2012), and neurons tend to connect with remote neurons in a limited number of brain regions (Hooks et al., 2013; Oh et al., 2014; Denardo et al., 2015). The probability of connecting with a neighboring neuron decreases with the distance between neurons.

Given that the communication of spike information in the simulation on a distributed parallel computer has a significant computational cost, a parallelization method for efficient communication is detailed in the following sections.

## Results

### Tile Parallelization and Performance

In this section, we investigate the relationship between parallelization method and communication of spike information in layered neural sheets. If the properties of the neural sheets and the hardware specification of the computers meet some conditions, the tile partitioning method has three advantages for communication among compute nodes compared with random assignment of neurons to compute nodes. These advantages are (1) replacement of slow network communications with fast memory access, (2) reduction of communication data by placing postsynaptic neurons into neighboring tiles, and (3) a constant communication data amount per compute node using the point-to-point communication function. The details of the conditions and advantages are described in the following sections.

### Replacement of Slow Network Communication With Fast Memory Access

**FIGURE 7:**
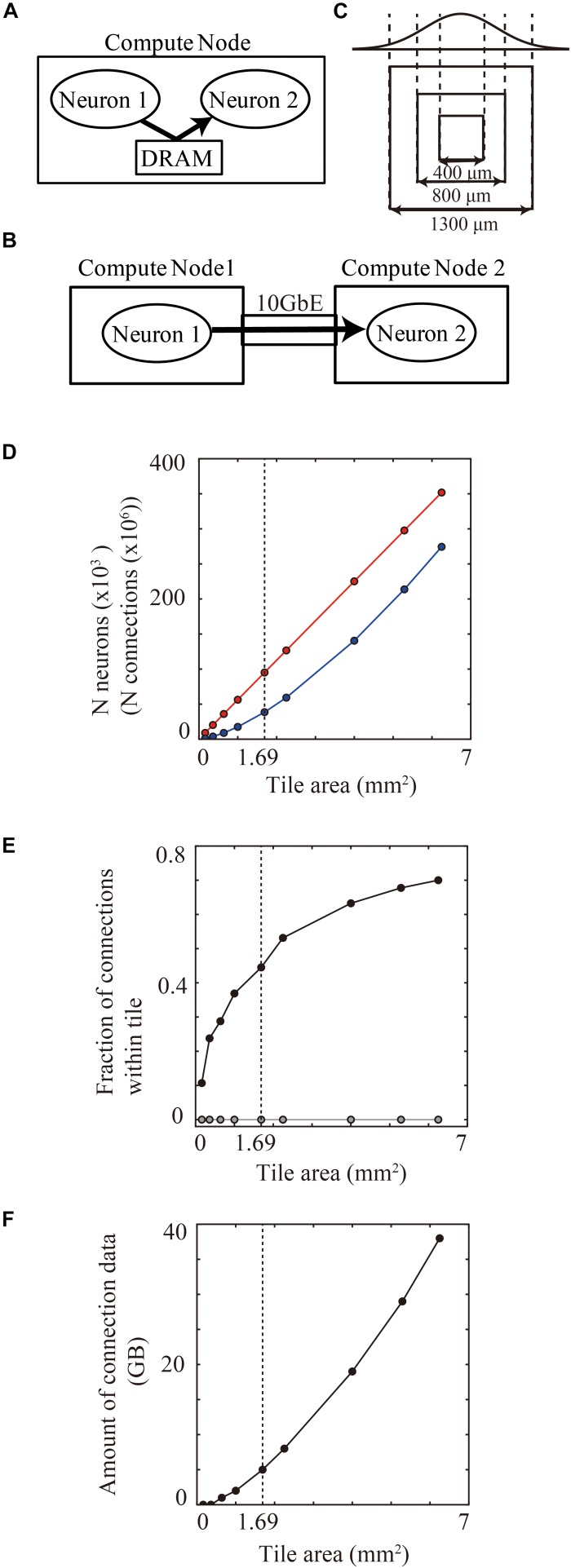
Communication of spike information within and across tiles. **(A)** Delivery of spike information between neuron1 and neuron2 through DRAM within the same compute node. **(B)** Delivery of spike information between neuron1 in compute node 1 and neuron2 in compute node 2 through gigabit ether. **(C)** Maximum spatial extent of connections in cortical layered sheet (upper) and different sizes of tiles (lower). The two standard deviation of the Gaussian function is 1300 μm. **(D)** The numbers of neurons (red) and synapses (blue) per tile. **(E)** Fraction of connections that both presynaptic and postsynaptic neurons are located within one compute tile in tile partitioning method (black). Gray line shows the theoretical fraction in the worst-case scenario that neurons are randomly distributed among 100,000 compute nodes. **(F)** Amount of memory consumption of connections per tile. In **(D–F)**, vertical dotted lines denote 1.69 mm^2^ (1300 μm × 1300 μm).

When one compute node includes a pair of connected presynaptic and postsynaptic neurons, a DRAM on the compute node can allow fast transfer of spike information between the pair of neurons (Figure 7A). When the presynaptic and postsynaptic neurons are assigned to two distinct compute nodes, network communication hardware such as a 10 gigabit ether network performs a slower transfer (Figure 7B). Therefore, the positioning of presynaptic and postsynaptic neurons in the same compute node has an advantage for spike data transfer.

When neurons are randomly assigned to compute nodes, irrespective of the spatial positions of the neurons, the proportion of connected presynaptic and postsynaptic neurons within one compute node depends on the total numbers of compute nodes. For example, when a simulation runs on the maximum number of 82,944 compute nodes in the K computer, the proportion becomes 0.0012% (1/the numbers of compute nodes).

In the tile partitioning method, the proportion of connected presynaptic and postsynaptic neurons within one compute node depends upon the tile size. A tile tends to contain pairs of presynaptic and postsynaptic neurons more frequently when there are dense connections between neighboring neurons, as described in see section “Local-Dense and Remote-Sparse Connections and Parallelization”. The proportion of inclusion of connected presynaptic and postsynaptic neurons within one tile thus depends upon tile size and the spatial extent of the connections (Figure 7C). The number of neurons and synapses increases linearly as the area of the tile size increases (Figure 7D). Figure 7E shows the proportional increase with increase in the tile size in the layered cortical sheet. At 1.69 mm^2^ (1.3 × 1.3 mm^2^), one tile had 95,124 neurons and 360 million connections (Figure 7D) and consumed 5.03 GB for all connections (Figure 7F). In total connections per compute node, 47.4% (170 million) of the connections contained both presynaptic and postsynaptic neurons in the same tile.

These results suggest that modern supercomputers consisting of compute nodes with more than 10 GB memory can benefit from the tile-partition method by replacing slow network communication with fast memory access.

### Reduction of Amount of Communication Data by Placing Postsynaptic Neurons Together in Neighbor Tiles

**FIGURE 8:**
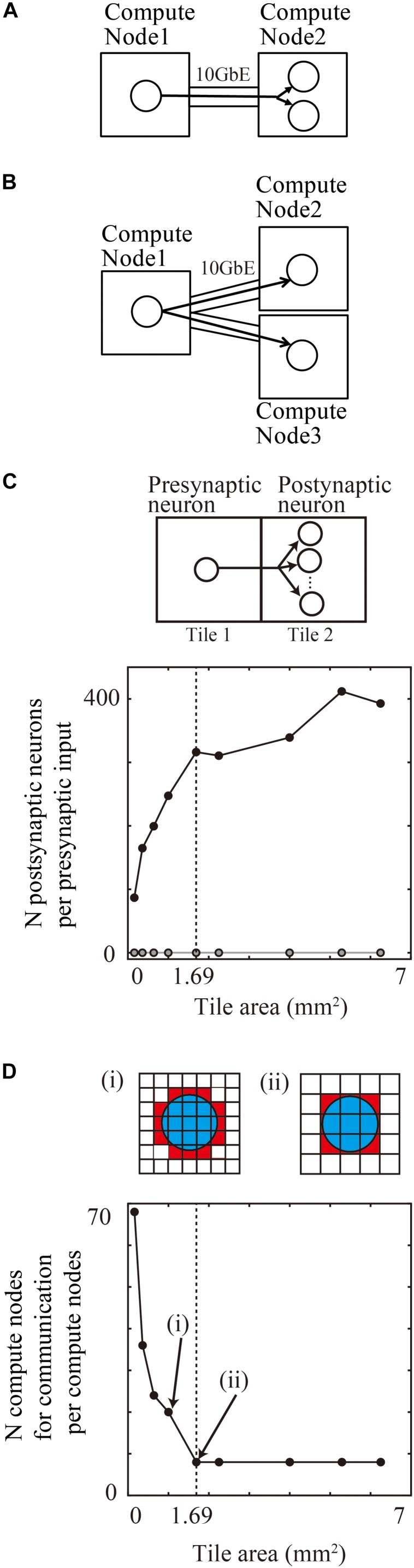
Communication reduction for shared input by postsynaptic neurons in the same tile. **(A)** Delivery of spike information from one presynaptic neuron in one compute node to two postsynaptic neurons in the other compute node. **(B)** Delivery of spike information from one presynaptic neuron in one compute node to two postsynaptic neurons in the other two compute nodes. **(C)** The average numbers of postsynaptic neurons per presynaptic input from the outside tile. **(D)** The number of tiles with which one tile communicates spike information. (i) and (ii) show the spatial extent of connections (blue) and connected tiles (brown) and not connected tiles (white). Small tiles in (i) communicate with more tiles than the large tiles in (ii).

When a presynaptic neuron assigned to one compute node projects to two postsynaptic neurons assigned to another compute node, the first compute node sends spike information about the presynaptic neuron to the other compute node (Figure 8A). When the two postsynaptic neurons are assigned to two different compute nodes, the first compute node, with the presynaptic neuron, sends the spike information to the other two compute nodes (Figure 8B). This means that collecting postsynaptic neurons with the same incoming signal into a single compute node decreases the total amount of communication data. Furthermore, the placement of postsynaptic neurons in the same compute node reduces the number of the compute nodes with the postsynaptic neurons sharing the input. This placement results in a decrease in the number of communication function calls if point-to-point communication is used in parallel computing.

In a realistic simulation of cortical neurons with thousands of projections on tens of thousands of compute nodes, the placement of postsynaptic neurons in the same compute nodes reduced the requirement for communication.

When neurons are randomly assigned to compute nodes irrespective of the spatial positions of neurons, the proportion of postsynaptic neurons sharing inputs from outside tiles depends upon the total number of compute nodes.

For example, when a simulation runs on the maximum number of 82,944 compute nodes of the K computer, 0.0012% (1/the numbers of compute nodes) of the postsynaptic neurons with the same input are located in the same compute node. It is therefore rare that more than two postsynaptic neurons share an input in a model of layered cortical sheets (the numbers of connections per presynaptic neurons/the numbers of compute nodes).

In the tile partitioning of neural sheets, postsynaptic cells receiving the same projection tend to be located in the same tile because of the preservation of local circuit structure. Figure 8C shows the average number of postsynaptic cells receiving the same input from a presynaptic cell outside the tile. Although the number tends to increase with increases in size, it is not a simple monotonic increase. Until the maximum spatial extent of 1300 μm, the numbers increase because any position in the tile receives connections from all of the neighboring tiles. When the tile size became larger than the maximum connection length, the edge part does not receive connections from the opposite side of the tiles and the density of connections from neighboring tiles decreases. The number saturates when the tile size is twice that of the maximum spatial extent of connections. At a tile size 1.69 mm^2^ (1.3 × 1.3 mm), the average numbers of postsynaptic cells receiving the same input is 316.3 (*n* = 598,474 presynaptic neurons from eight neighboring tiles). This result means that the amount of communication data can be decreased by a factor of 316.3 compared with the worst case, in which postsynaptic neurons do not share the same input due to the random connections.

Figure 8D shows the number of compute nodes from which one compute node receives spike information, which decreases with an increase in the tile size. At a tile size of 1.69 mm^2^ (1.3 × 1.3 mm), the number of tiles was eight, when the tiles communicate only with the nearest neighbor tiles.

These results show that the tile size used in the tile partitioning method on modern supercomputers has advantages due to the reduction in communication data and numbers of compute nodes in point-to-point communication.

### Constant Communication Data Amount per Compute Node in Point-to-Point Communication

If the tile size is sufficiently larger than spatial extent of connections, only neighboring tiles communicate spike information each other. Then, the amount of communication data is constant per tile, irrespective of the total area of the layered neural sheet.

The average amount of data for sending and receiving was 85 MB per compute node for 1 s of biological time when one comute node communicated with the neighororing tiles for 1.69 mm^2^ of the tile size and the neurons fired at around 4 Hz. The amount of communication data of different tiles did not differ, except for the tiles on the edge of the neural sheet. This result suggests that the tile partitioning method with the point-to-point communication kept constant communication data amount per compute node which has an advantage for scaling up the size of layered sheet models.

### Scaling Performance

To investigate whether the advantages of the tile partitioning method described in the previous section actually works in large-scale simulation, we tested the scaling performance of the layered cortical sheet model using the MONET on the K computer.

**FIGURE 9:**
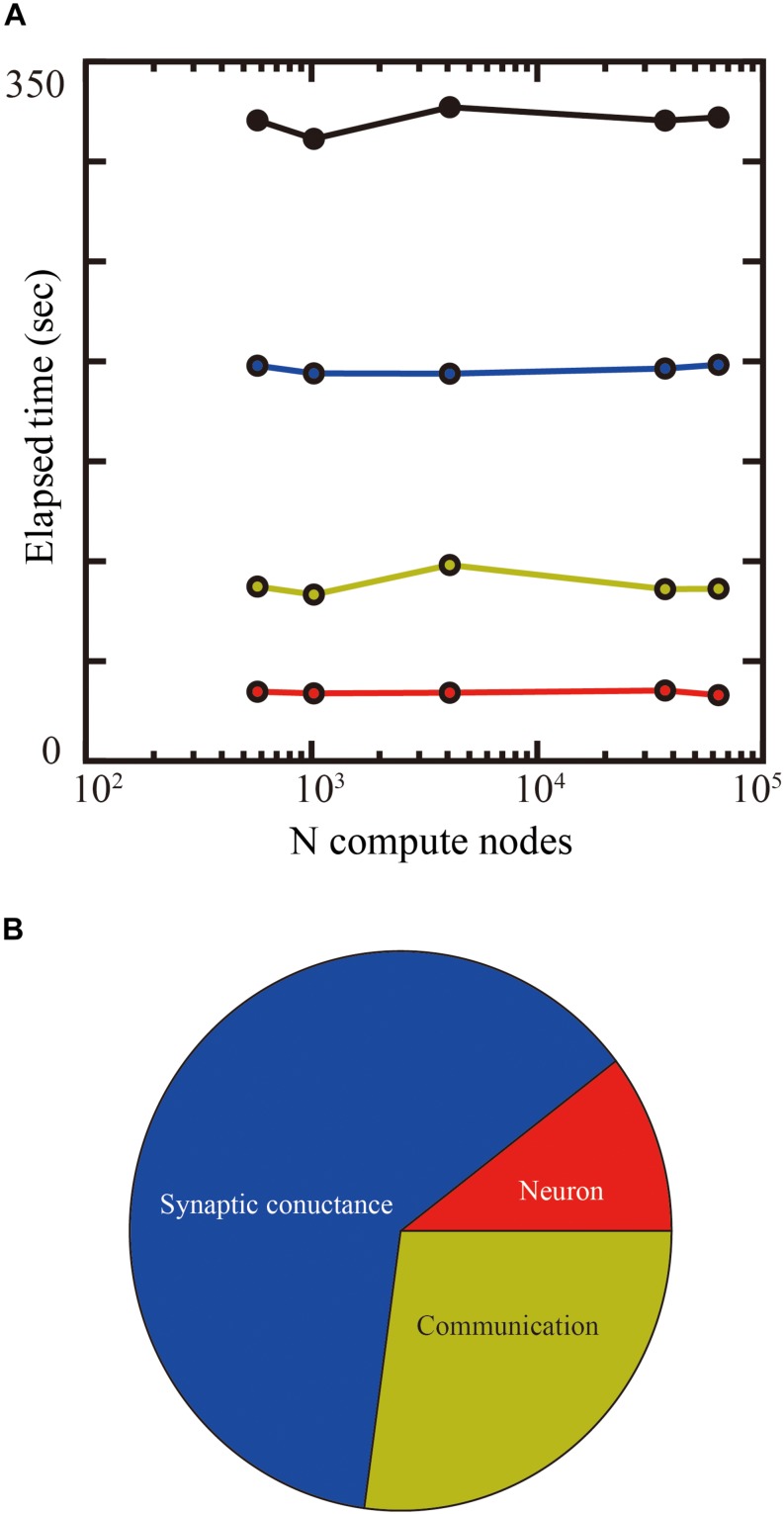
Weak-scaling performance test of the layered cortical sheet model on the K computer. **(A)** Elapsed times of simulation for 1 s of biological time. Total elapsed time (black), calculation of neurons (red), synapses (blue), and communication (yellow). **(B)** Breakdown of computational time using 63,504 compute nodes. The color code is as in **(A)**.

Up to 14 GB of DRAM per compute node is available for users on the K computer, so we used 1.69 mm^2^ (1.3 × 1.3 mm) of tiles in the tile partitioning method. We investigated the weak-scaling performance of the layered cortical sheet using from 576 to 63,504 compute nodes on the K computer. Biological time in the simulation was 1 s. Figure 9A shows the result of weak-scaling performance test. In the weak scaling performance test, computational performance is tested for fixed model size per compute node while varying the numbers of compute nodes and total model size. We assigned 1.69 mm^2^ (1.3 × 1.3 mm) of tiles to one compute node. The elapsed time did not change materially with an increase in the numbers of compute nodes. The processing times of the neurons, synaptic conductance, and communication also did not change significantly. The largest scale, executed by 63,504 compute nodes, achieved a simulation of 1,073 cm^2^ of layered cortical sheet with 6.04 billion neurons and 24.5 trillion connections.

Figure 9B shows the breakdown of elapsed time in simulation for 1 s of biological time. The percentages of calculation used by synaptic conductance, calculation of neurons, and communications were 62.4, 10.4, and 27.1%, respectively.

## Discussion

In the current study, we investigated an efficient parallelization method for spiking neural network models with a single-compartment neuron model as a step toward the development of whole brain models.

First, we summarized key physiological features of the whole brain in terms of the numbers of neurons and synapses, circuit structure, and connections in the brain. The three key features are (1) dominance of the cortex and cerebellum in terms of the numbers of neurons and synapses; (2) a layered sheet structure in the dominant regions, cortex and cerebellum; and (3) locally dense and remotely sparse connections in the brain.

Given these three key features, we examined the effectiveness of tile partitioning method for the layered cortical sheet model in terms of communication. Assuming the use of computers with 10 GB of memory per compute node, we showed that the tile partitioning method in the layered cortical sheet replaced about half of the slow communication with fast memory access. In addition, the tile partitioning method produced a reduction in the amount of communication data among tiles by a factor of 316.3 compared with random assignment of cortical neurons to 100 thousand compute nodes. The tile partitioning method also reduced the number of tiles involved in communication to neighboring tiles, which results in the reduction of the number of communication function calls.

Finally, weak-scaling performance was tested using 576 to 63,504 compute nodes. Use of the maximum number of compute nodes achieved a simulation of a layered cortical sheet with 6.04 billion neurons and 24.5 trillion connections.

In this study, we investigated the proposed method for layered cortical sheet using the parameters of the mouse primary motor cortex. The proposed method may work for simulation of the other cortical areas due to the similarity of the neural density and circuit structure over the cortical sheet. In the accompany paper (Yamaura et al., in review), we reported that the proposed method showed good scaling performance for simulation of a layered cerebellar sheet that has larger neural density and lower numbers of connections per a tile. The proposed method may be effective for brain regions with repeated structure, such as striatum in the basal ganglia.

Pre- and post-processes for whole brain simulation become huge for modeling and analyzing different brain regions, such as introduction of connectome or comparison with multimodal experimental dataset. Execution of simulation and pre- and post processing all at once on supercomputers might be practical solution.

An amount of computation of synaptic conductance and communication data of spike information depends on the firing rates in the network. We tested computational performance of the proposed method for a resting state of the model with about 4 Hz of a mean firing rate for 1 s. The firing rate does not change with time substantially as long as initial conditions of neuron models are set in a biologically plausible range. If more than 1 s of biological time is used, the wall-clock time increases linearly with the biological time. On the other hand, firing rates of different cortical regions become high or low in an active state depending on whether it is default-mode network or not (Broyd et al., 2009). The dynamic change can cause load imbalance among tiles in parallelization. Although global average of the firing rate may be kept to a similar range as estimated in the report of constant energy consumption (Kiviniemi et al., 2003), we will need further investigation of influence of the dynamically changing neural activity on load balancing of a cortical model.

### Typical Hardware Specification and Tile Partitioning Method

In respect to the reduction of communication in the tile partitioning method, two hardware features in the current supercomputers are tightly related to the performance.

The first hardware feature is an amount of memory per compute node. About 10 GB per compute node can implement 1–2 mm^2^ of cortical tile, which covers a large part of the spatial extent of the intra-regional connections of the cortex in the horizontal direction. The memory amount per compute node has been increasing over years according to Moore’s law. The phenomenon may improve the efficiency of communication by enlarging the achievable cortical tile area in the tile partitioning method.

The second hardware feature is the number of compute nodes in supercomputers. The typical number of compute nodes ranges from several thousand to 100 thousand. For example, the Summit (Oak Ridge National Laboratory, United States) has 4,608 compute nodes, the Sierra (Lawrence Livermore National Laboratory, United States) has 4,320, the Sunway TaifuLight (National Supercomputing Center China) has 40,960, and the K computer (RIKEN Center for Computational Science, Japan) has 82,944 nodes. The use of multiple compute nodes leads to neurons being distributed between nodes, with a concomitant need for communication of information about spikes between the compute nodes. It is necessary to take this issue into account when using current supercomputers.

### Difference of the Brain Among Animal Spices

In the current study, the layered cortical sheet model was developed on the basis of the physiological parameters of the mouse primary motor cortex. The tile partitioning method should work with the other parameters of mammalian cortex, because the layered sheet structure of the cortex is conserved among different mammalian brains (Defelipe, 2011). Even if the total numbers of neurons and volume differ by a factor of 1,000 among mammals, layer structure, layer thickness, neural density, and number of synapses per neuron falls within the range of one order of magnitude. For example, the numbers of neurons and the synapses per unit area of a cortical sheet range within a factor of 2.5 between mouse and human (Defelipe, 2011). In particular, primates have larger numbers of synapses per neuron than rodents, which may require relatively large communication per cortical sheet area. Efficient communication performance in the tile partitioning method may work in the simulation of the mammalian cortex including primate and rodent cortex.

### Future Works Toward Whole Brain Simulation

In the current study, we focused on the simulation of a homogeneous single brain region with only intra-regional connections. To implement a whole brain simulation of multiple, heterogeneous brain regions, it is necessary to establish an efficient way to implement inter-regional connections and a mechanism for load balancing among the different brain regions.

The implementation of inter-regional connections can be developed by extending intra-regional connections using signal transmission delay in the current study. Using the tile-partition method, a specific communication interval can be set for each pair of tiles. Longer signal transmission delays between tiles allow tiles to communicate at longer intervals. In terms of memory consumption, additional memory allocation is required for the implementation of the inter-regional connections. A similar level of memory allocation to intra-regional connections may be needed for the inter-regional connections in the whole brain simulation.

In terms of load balancing among different brain regions, there are two possible solutions. One is to assign different compute nodes to different brain regions according to their computational load. Another solution is to make a sheet consisting of different regions of sheets taking into account inter-regional structure such as the cortico–thalamo–cerebellar loop circuit.

### Proposed Method for GPU-Based and NMC-Based Supercomputers

Graphic processing units (GPUs) and neuromorphic chips (NMCs) are promising technology for accelerating calculation of spiking neural networks. Although we demonstrated effectiveness of the proposed method using a CPU-based supercomputer in the current study, the proposed method should be applicable to neural network simulations on GPU- based and NMC-based supercomputers.

In the application of the proposed method to GPU- based supercomputers which contain CPUs as a host computer, calculations of membrane potentials and synaptic inputs can be offloaded to GPUs without changing communication part by CPUs. As the proposed method in the current study, calculation by GPUs and communication by CPUs can be overlapped. The assignment may increase computational efficiency of GPUs which are workhorses in GPU-based supercomputers.

For NMC-based supercomputers, the proposed method can be used for parallelization of spiking neural networks and designing a communication way among the NMCs with asynchronous point-to-point communications. However, the degree of the effectiveness of the proposed method on the NMCs depends on the numbers of neurons which the NMC chips can implement per one chip and spatial extents of connections of target neural networks, as examined in chapters 4.2 and 4.3 (Figures 7, 8). The recent representative NMCs, such as SpiNNaker (16 thousand neurons per a chip, Furber et al., 2013), BrainSacleS (200 thousand neurons per a wafer module, Schemmel et al., 2010) and TrueNorth (one million neurons per a chip, Merolla et al., 2014) have a potential to benefit from the proposed method.

The use of proposed method on CPUs and GPUs has advantages over NMCs in terms of flexibility for changing configuration of neural networks and adjusting details of the parallelization way. On the other hand, the NMCs are superior to CPUs and GPUs in an energy efficient computing of specific neural networks that fit with the configuration of NMCs. These should be used properly according to their targets.

### Whole Brain Simulation Scale Simulation on the Next Generation Supercomputers

The current study suggested the possibilities for further scaling up the layered cortical sheet model. The human cortex has 16 billion neurons in 2500 cm^2^ of the cortical sheet (Herculano-Houzel, 2009). The current study performed a simulation with 6.04 billion neurons in 1073 cm^2^ of the cortical sheet corresponding to more than one-third of the human cortex. To implement a simulation of the full human cortex, a model which is three times the size of the current model will be needed. As shown in the companying report, models of layered cerebellar sheets at a realistic human scale are ready. For simultaneous simulation of the cerebellar sheet and the three-times-larger cortical sheet, more than four times the amount of computational resources will be required.

The next generation of supercomputers in 2020s will have approximately 100 times greater computational performance than the K computer. These exascale computers will be able to implement realistic spiking neural network simulations of the whole brain. The successor of the K computer has already been announced as being available around 2021. According to the announcement, it will have more than 150 thousand compute nodes, each of which has 48-core CPUs and 32 GB DRAM.

The amount of memory will enable the implementation of tile areas twice the size of current tiles per compute node using the tile partitioning method and could improve communication performance by increasing the fraction of inclusion of presynaptic and postsynaptic neurons in the same compute node (Figure 7E).

The performance gain in communication in the next generation supercomputers (× 2) will be much smaller than those of computation (× 20) and memory (× 10), which might result in difficulty because of slower communication. To balance the performance, the efficient communication of spike information in the current study may help efficient whole brain simulation using exascale computers.

## Data Availability Statement

The datasets generated for this study are available on request to the corresponding author. The binary executable files of the MONET are available at https://github.com/junigarashi/MONET.

## Author Contributions

JI designed the study, developed the network simulation software, and wrote the manuscript with the help of HY and TY. JI and HY performed the simulations. JI, HY, and TY analyzed and interpreted the simulated data.

## Conflict of Interest

The authors declare that the research was conducted in the absence of any commercial or financial relationships that could be construed as a potential conflict of interest.

## References

[B1] Ajima Y. Kawashima T. Okamoto T. Shida N. Hirai K. Shimizu T. (2018). “The tofu interconnect D,” in *Proceedings of the IEEE Int. Conf. Clust. Comput. ICCC 2018-September*, (Belfast: IEEE), 646–654.

[B2] Ananthanarayanan R. Esser S. K. Simon H. D. Modha D. S. (2009). The cat is out of the bag: cortical simulations with 109 neurons, 1013 synapses. in proceedings of the conference on high performance computing networking. *Storage Anal.* 63 1–12.

[B3] Apicella A. J. Wickersham I. R. Seung H. S. Shepherd G. M. (2012). Laminarly orthogonal excitation of fast-spiking and low-threshold-spiking interneurons in mouse motor cortex. *J. Neurosci.* 32 7021–7033. 10.1523/JNEUROSCI.0011-12.2012 22593070 PMC3377057

[B4] Avermann M. Tomm C. Mateo C. Gerstner W. Petersen C. C. (2012). Microcircuits of excitatory and inhibitory neurons in layer 2/3 of mouse barrel cortex. *J. Neurophysiol.* 107 3116–3134. 10.1152/jn.00917.2011 22402650

[B5] Broyd S. J. Demanuele C. Debener S. Helps S. K. James C. J. Sonuga-Barke E. J. S. (2009). Default-mode brain dysfunction in mental disorders: a systematic review. *Neurosci. Biobehav. Rev.* 33 279–296. 10.1016/j.neubiorev.2008.09.002 18824195

[B6] Buckner R. L. Krienen F. M. Castellanos A. Diaz J. C. Yeo B. T. (2011). The organization of the human cerebellum estimated by intrinsic functional connectivity. *J. Neurophysiol.* 106 2322–2345. 10.1152/jn.00339.2011 21795627 PMC3214121

[B7] Carnevale N. T. Hines M. L. (2006). *The NEURON Book.* Cambridge: Cambridge University Press.

[B8] Defelipe J. (2011). The evolution of the brain, the human nature of cortical circuits, and intellectual creativity. *Front. Neuroanat.* 5:29. 10.3389/fnana.2011.00029 21647212 PMC3098448

[B9] Deisseroth K. (2015). Optogenetics: 10 years of microbial opsins in neuroscience. *Nat. Neurosci.* 18 1213–1225. 10.1038/nn.4091 26308982 PMC4790845

[B10] Denardo L. A. Berns D. S. Deloach K. Luo L. (2015). Connectivity of mouse somatosensory and prefrontal cortex examined with trans-synaptic tracing. *Nat. Neurosci.* 18 1687–1697. 10.1038/nn.4131 26457553 PMC4624522

[B11] Eccles J. C. Ito M. Szentàgothai J. (1967). *The Cerebellum as a Neuronal Machine.* New York, NY: Springer-Verlag.

[B12] Fino E. Yuste R. (2011). Dense inhibitory connectivity in neocortex. *Neuron* 69 1188–1203. 10.1016/j.neuron.2011.02.025 21435562 PMC3086675

[B13] Furber S. B. Lester D. R. Plana L. A. Garside J. D. Painkras E. Temple S. (2013). Overview of the SpiNNaker system architecture. *IEEE Trans. Comput.* 62 2454–2467. 10.1109/TC.2012.142

[B14] Gewaltig M. O. Diesmann M. (2007). NEST (NEural Simulation Tool). *Scholarpedia* 2:1430 10.4249/scholarpedia.1430

[B15] Glasser M. F. Smith S. M. Marcus D. S. Andersson J. L. Auerbach E. J. Behrens T. E. (2016). The human connectome project’s neuroimaging approach. *Nat. Neurosci.* 19 1175–1187. 10.1038/nn.4361 27571196 PMC6172654

[B16] Helias M. Kunkel S. Masumoto G. Igarashi J. Eppler J. M. Ishii S. (2012). Supercomputers ready for use as discovery machines for neuroscience. *Front. Neuroinform.* 6:26. 10.3389/fninf.2012.00026 23129998 PMC3486988

[B17] Herculano-Houzel S. (2009). The human brain in numbers: a linearly scaled-up primate brain. *Front. Hum. Neurosci.* 3:31. 10.3389/neuro.09.031.2009 19915731 PMC2776484

[B18] Hooks B. M. Hires S. A. Zhang Y. X. Huber D. Petreanu L. Svoboda K. (2011). Laminar analysis of excitatory local circuits in vibrissal motor and sensory cortical areas. *PLoS Biol.* 9:e1000572. 10.1371/journal.pbio.1000572 21245906 PMC3014926

[B19] Hooks B. M. Mao T. Gutnisky D. A. Yamawaki N. Svoboda K. Shepherd G. M. (2013). Organization of cortical and thalamic input to pyramidal neurons in mouse motor cortex. *J. Neurosci.* 33 748–760. 10.1523/JNEUROSCI.4338-12.2013 23303952 PMC3710148

[B20] Hunnicutt B. J. Long B. R. Kusefoglu D. Gertz K. J. Zhong H. Mao T. (2014). A comprehensive thalamocortical projection map at the mesoscopic level. *Nat. Neurosci.* 17 1276–1285. 10.1038/nn.3780 25086607 PMC4152774

[B21] Igarashi J. Shouno O. Fukai T. Tsujino H. (2011). Real-time simulation of a spiking neural network model of the basal ganglia circuitry using general purpose computing on graphics processing units. *Neural Netw.* 24 950–960. 10.1016/j.neunet.2011.06.008 21764258

[B22] Izhikevich E. M. Edelman G. M. (2008). Large-scale model of mammalian thalamocortical systems. *Proc. Natl. Acad. Sci. U.S.A.* 105 3593–3598. 10.1073/pnas.0712231105 18292226 PMC2265160

[B23] Izhikevich E. M. Gally J. A. Edelman G. M. (2004). Spike-timing dynamics of neuronal groups. *Cereb. Cortex* 14 933–944. 10.1093/cercor/bhh053 15142958

[B24] Jiang X. Wang G. Lee A. J. Stornetta R. L. Zhu J. J. (2013). The organization of two new cortical interneuronal circuits. *Nat. Neurosci.* 16 210–218. 10.1038/nn.3305 23313910 PMC3589105

[B25] Jin X. Furber S. B. Woods J. V. (2008). “Efficient modelling of spiking neural networks on a scalable chip multiprocessor,” in *In Proceedings of the 2008 IEEE International Joint Conference on Neural Networks (IEEE World Congress on Computational Intelligence)*, (Piscataway, NJ: IEEE), 2812–2819.

[B26] Jordan J. Ippen T. Helias M. Kitayama I. Sato M. Igarashi J. (2018). Extremely Scalable spiking neuronal network simulation code: from laptops to exascale computers. *Front. Neuroinform.* 12:2. 10.3389/fninf.2018.00034 29503613 PMC5820465

[B27] Kätzel D. Zemelman B. V. Buetfering C. Wölfel M. Miesenböck G. (2011). The columnar and laminar organization of inhibitory connections to neocortical excitatory cells. *Nat. Neurosci.* 14 100–107. 10.1038/nn.2687 21076426 PMC3011044

[B28] Kiviniemi V. Kantola J. H. Jauhiainen J. Hyvärinen A. Tervonen O. (2003). Independent component analysis of nondeterministic fMRI signal sources. *Neuroimage* 19(2 pt 1), 253–260. 10.1016/S1053-8119(03)00097-91 12814576

[B29] Kozloski J. Wagner J. (2011). An ultrascalable solution to large-scale neural tissue simulation. *Front. Neuroinform.* 5:15. 10.3389/fninf.2011.00015 21954383 PMC3175572

[B30] Kunkel S. Schmidt M. Eppler J. M. Plesser H. E. Masumoto G. Igarashi J. (2014). Spiking network simulation code for petascale computers. *Front. Neuroinform.* 8:78. 10.3389/fninf.2014.00078 25346682 PMC4193238

[B31] Lee A. J. Wang G. Jiang X. Johnson S. M. Hoang E. T. Lanté F. (2015). Canonical organization of layer 1 neuron-led cortical inhibitory and disinhibitory interneuronal circuits. *Cereb. Cortex* 25 2114–2126. 10.1093/cercor/bhu020 24554728 PMC4494026

[B32] Lefort S. Tomm C. Floyd Sarria J. C. Petersen C. C. (2009). The excitatory neuronal network of the C2 barrel column in mouse primary somatosensory cortex. *Neuron* 61 301–316. 10.1016/j.neuron.2008.12.020 19186171

[B33] Lev D. L. White E. L. (1997). Organization of pyramidal cell apical dendrites and composition of dendritic clusters in the mouse: emphasis on primary motor cortex. *Eur. J. Neurosci.* 9 280–290. 10.1111/j.1460-9568.1997.tb01398.x 9058048

[B34] Markram H. Muller E. Ramaswamy S. Reimann M. W. Abdellah M. Sanchez C. A. (2015). Reconstruction and simulation of neocortical microcircuitry. *Cell* 163 456–492. 10.1016/j.cell.2015.09.029 26451489

[B35] Marsaglia G. (2015). Xorshift RNGs. *J. Stat. Softw.* 8 1–6.

[B36] Merolla P. A. Arthur J. V. Alvarez-Icaza R. Cassidy A. S. Sawada J. Akopyan F. (2014). A million spiking-neuron integrated circuit with a scalable communication network and interface. *Science* 345 668–673. 10.1126/science.1254642 25104385

[B37] Message Passing Interface Forum (2009). *MPI: A Message-Passing Interface Standard Version* 2.2. Knoxville, TX: Tech rep.

[B38] Miyazaki H. Kusano Y. Shinjou N. Fumiyoshi S. Yokokawa M. Watanabe T. (2012). Overview of the K computer system. *Fujitsu Sci. Tech. J.* 48 255–265.

[B39] Moren J. Igarashi J. Shouno O. Yoshimoto J. Doya K. (2019). “Dynamics of basal ganglia and thalamus in Parkinsonian tremor,” in *Multiscale Models of Brain Disorders*, ed. Cutsuridis V. (New York, NY: Springer-Verlag).

[B40] Oh S. W. Harris J. A. Ng L. Winslow B. Cain N. Mihalas S. (2014). A mesoscale connectome of the mouse brain. *Nature* 508 207–214. 10.1038/nature13186 24695228 PMC5102064

[B41] OpenMP Architecture Review Board (2008). *OpenMP Application Program Interface version 3.0.* Available at: http://www.openmp.org/wp-content/uploads/spec30.pdf

[B42] Packer A. M. Yuste R. (2011). Dense, unspecific connectivity of neocortical parvalbumin-positive interneurons: a canonical microcircuit for inhibition? *J. Neurosci.* 31 13260–13271. 10.1523/JNEUROSCI.3131-11.2011 21917809 PMC3178964

[B43] Pala A. Petersen C. C. H. (2015). In vivo measurement of cell-type-specific synaptic connectivity and synaptic transmission in layer 2/3 mouse barrel cortex. *Neuron* 85 68–75. 10.1016/j.neuron.2014.11.025 25543458 PMC4305188

[B44] Parvizi J. Kastner S. (2018). Promises and limitations of human intracranial electroencephalography. *Nat. Neurosci.* 21 474–483. 10.1038/s41593-018-0108-2 29507407 PMC6476542

[B45] Pesaran B. Vinck M. Einevoll G. T. Sirota A. Fries P. Siegel M. (2018). Investigating large-scale brain dynamics using field potential recordings: analysis and interpretation. *Nat. Neurosci.* 21 903–919. 10.1038/s41593-018-0171-8 29942039 PMC7386068

[B46] Pfeffer C. K. Xue M. He M. Huang Z. J. Scanziani M. (2013). Inhibition of inhibition in visual cortex: the logic of connections between molecularly distinct interneurons. *Nat. Neurosci.* 16 1068–1076. 10.1038/nn.3446 23817549 PMC3729586

[B47] Rotter S. Diesmann M. (1999). Exact digital simulation of time-invariant linear systems with applications to neuronal modeling. *Biol. Cybern.* 81 381–402. 10.1007/s004220050570 10592015

[B48] Schemmel J. Brüderle D. Grübl A. Hock M. Meier K. Millner S. (2010). “A wafer-scale neuromorphic hardware system for large-scale neural modeling,” in *ISCAS 2010 - 2010 IEEE International Symposium on Circuits and Systems: Nano-Bio Circuit Fabrics and Systems*, (Paris: IEEE).

[B49] Shepherd G. M. (2013). Corticostriatal connectivity and its role in disease. *Nat. Rev. Neurosci.* 14 278–291. 10.1038/nrn3469 23511908 PMC4096337

[B50] Song S. Sjöström P. J. Reigl M. Nelson S. Chklovskii D. B. (2005). Highly nonrandom features of synaptic connectivity in local cortical circuits. *PLoS Biol.* 3:e68. 10.1371/journal.pbio.0030068 15737062 PMC1054880

[B51] Standring S. (2016). *Gray’s Anatomy 41th Edition: The Anatomical Basis of Clinical Practice.* New York, NY: Elsevier Limited.

[B52] Tremblay R. Lee S. Rudy B. (2016). GABAergic interneurons in the neocortex: from cellular properties to circuits. *Neuron* 91 260–292. 10.1016/j.neuron.2016.06.033 27477017 PMC4980915

[B53] Weiler N. Wood L. Yu J. Solla S. A. Shepherd G. M. (2008). Top-down laminar organization of the excitatory network in motor cortex. *Nat. Neurosci.* 11 360–366. 10.1038/nn2049 18246064 PMC2748826

[B54] Xu X. Callaway E. M. (2009). Laminar specificity of functional input to distinct types of inhibitory cortical neurons. *J. Neurosci.* 29 70–85. 10.1523/JNEUROSCI.4104-08.2009 19129386 PMC2656387

[B55] Xue M. Atallah B. V. Scanziani M. (2014). Equalizing excitation-inhibition ratios across visual cortical neurons. *Nature* 511 596–600. 10.1038/nature13321 25043046 PMC4117808

[B56] Yamazaki T. Igarashi J. (2013). Realtime cerebellum: a large-scale spiking network model of the cerebellum that runs in realtime using a graphics processing unit. *Neural. Netw.* 47 103–111. 10.1016/j.neunet.2013.01.019 23434303

[B57] Yamazaki T. Igarashi J. Makino J. Ebisuzaki T. (2019). Real-time simulation of a cat-scale artificial cerebellum on PEZY-SC processors. *Int. J. High Perf. Com. App.* 33 155–168. 10.1177/1094342017710705

[B58] Yeo B. T. Krienen F. M. Sepulcre J. Sabuncu M. R. Lashkari D. Hollinshead M. (2011). The organization of the human cerebral cortex estimated by intrinsic functional connectivity. *J. Neurophysiol.* 106 1125–1165. 10.1152/jn.00338.2011 21653723 PMC3174820

[B59] Zingg B. Hintiryan H. Gou L. Song M. Y. Bay M. Bienkowski M. S. (2014). Neural networks of the mouse neocortex. *Cell* 156 1096–1111. 10.1016/j.cell.2014.02.023 24581503 PMC4169118

